# Role of Gaze Cues in Interpersonal Motor Coordination: Towards Higher Affiliation in Human-Robot Interaction

**DOI:** 10.1371/journal.pone.0156874

**Published:** 2016-06-09

**Authors:** Mahdi Khoramshahi, Ashwini Shukla, Stéphane Raffard, Benoît G. Bardy, Aude Billard

**Affiliations:** 1 Learning Algorithms and Systems Laboratory, School of Engineering, EPFL, Lausanne, Switzerland; 2 University Department of Adult Psychiatry, CHRU, & Laboratory Epsylon, EA 4556, Montpellier, France; 3 Movement to Health Laboratory, EuroMov, Montpellier-1 University, Montpelier, France; 4 Institut Universitaire de France, Paris, France; Defence Science and Technology Group, AUSTRALIA

## Abstract

**Background:**

The ability to follow one another’s gaze plays an important role in our social cognition; especially when we *synchronously* perform tasks together. We investigate how gaze cues can improve performance in a simple coordination task (i.e., the *mirror game*), whereby two players mirror each other’s hand motions. In this game, each player is either a leader or follower. To study the effect of gaze in a systematic manner, the leader’s role is played by a robotic avatar. We contrast two conditions, in which the avatar provides or not explicit gaze cues that indicate the next location of its hand. Specifically, we investigated (a) whether participants are able to exploit these gaze cues to improve their coordination, (b) how gaze cues affect action prediction and temporal coordination, and (c) whether introducing active gaze behavior for avatars makes them more realistic and human-like (from the user point of view).

**Methodology/Principal Findings:**

43 subjects participated in 8 trials of the mirror game. Each subject performed the game in the two conditions (with and without gaze cues). In this within-subject study, the order of the conditions was randomized across participants, and subjective assessment of the avatar’s realism was assessed by administering a post-hoc questionnaire. When gaze cues were provided, a quantitative assessment of synchrony between participants and the avatar revealed a significant improvement in subject reaction-time (RT). This confirms our hypothesis that gaze cues improve the follower’s ability to predict the avatar’s action. An analysis of the pattern of frequency across the two players’ hand movements reveals that the gaze cues improve the overall temporal coordination across the two players. Finally, analysis of the subjective evaluations from the questionnaires reveals that, in the presence of gaze cues, participants found it not only more human-like/realistic, but also easier to interact with the avatar.

**Conclusion/Significance:**

This work confirms that people can exploit gaze cues to predict another person’s movements and to better coordinate their motions with their partners, even when the partner is a computer-animated avatar. Moreover, this study contributes further evidence that implementing biological features, here task-relevant gaze cues, enable the humanoid robotic avatar to appear more human-like, and thus increase the user’s sense of affiliation.

## Introduction

The cooperative eye hypothesis [1] suggests that the visual characteristics of human eyes, such as scelra, iris, and pupil, evolved to make it easier to follow others’ gaze directions. According to this hypothesis, evolution enhances cooperative social interactions by providing a new social function; i.e., using gaze as a means to share one’s intention. A growing number of studies have investigated the use of gaze as a form of non-verbal communication in a variety of social interactions; e.g., to complement speech [2], and as a mechanism to orient others’ attention [3]. Gaze as a mean to orient other’s attention is possible if we can follow the gaze of others. The ability to follow other’s gaze-direction enables joint attention [4] that plays an important role in our social cognition [5]. Recent neurological studies have revealed visual cells sensitive to gaze direction [6]; these cells overlaps with neural mechanisms representing facial expression [7]. Moreover, eye contact modulates the activation of the social brain [8]. This suggests that the ability to generate and respond to gaze as a means of conveying intentions recruits common neural substrates [9, 10]. It has also been reported that gaze behavior is crucial for joint action [11, 12]. Orienting the gaze at the right location at the right time improves coordination with other individuals. It has been reported that gaze direction is also necessary in establishing a closed-loop dyadic interaction, which enables a better coordination in joint actions [13].

Social motor coordination as one aspect of social interaction, has received much interest in recent years; see [14] as a review. It refers to our ability to coordinate our movements with other individuals (i.e., interpersonal synchrony) to perform a task. The cognitive and socio-psychological aspects of joint action have been studied throughly; see [11] and [15]. Interpersonal synchrony provides an important foundation for social interaction, as it has been shown that the degree of interactional synchrony of bodily movements of co-actors during social interaction is a significant predictor of subsequent affiliation ratings and cooperation between individuals [16]. To better understand the mechanisms at the basis of joint action, cognitive and neural scientists have studied the underlying processes separately, including those responsible for joint attention [5], action observation/prediction [12, 17], action coordination [18], synchrony [19], and task sharing [20]. Moreover, the ability to follow another’s gaze is central to the joint action [13] via its roles in joint attention [21] and action observation [22].

In this work, we complement this body of literature and study the effect that gaze cues can have on dyadic interaction between a human and non-human partner, a computer generated avatar. Our main contribution is two-fold: First, using avatars’ systematic and structured behavior in a joint action, we provide a better understanding of human performance in joint action; second, we show that gaze behavior enable avatars to be effective partners in joint action. Specifically, we hypothesize that the avatars’ gaze can re-orient the attention of their human partners during the joint action for better coordination. We investigate how the avatar’s gaze cues might affect underlying cognitive processes in humans, such as action prediction and synchrony that can potentially lead to higher sense of realism.

To elaborate on the effects of gaze cues on dyadic interaction with avatars, we employed a simple framework that enables an in-depth investigation of synchronous coordination. The mirror game [23] is used to study motor coordination in dyadic interactions. In this game, individuals mirror one another’s hand movements with or without a designated leader. By measuring temporal coordination across hand trajectories, this game provides a framework for studying social coordination. Early results of the mirror game have provided a better understanding of the human ability for joint improvisation [23]. It has been shown that experts can create novel, synchronous, and confident (jitter-less) motions. Moreover, it helps to identify individual-specific signatures of motion that shape the behavior of the dyad [24]. Nonetheless, studying the behavior of the dyad makes it difficult to separate the individual contributions. In this study, we replace one player by an avatar, whose motion is structured and controlled explicitly. This enables us to attribute precisely the human’s contribution to the joint action and to have comparable experimental conditions. In addition, the human-avatar setting enables us to investigate the socio-psychological effects of avatars’ behaviors on human partners.

We are currently witnessing a growing number of applications for humanoid robots, androids, and computer simulated avatars in context of social interaction [25–27]. For instance, in telecommunication, androids can elicit a strong feeling of presence in the operator [27]. However, to enhance the human affiliation toward a robot or an avatar, researchers have tried to improve both the visual and behavioral aspects of android and avatars [28]. Among others, gaze behavior has been considered an effective element to enhance social interactions [29, 30]. It has been shown that by using gaze behavior, a robot can establish the participants’ roles in a conversational setting and increase the sense of affiliation among the individuals [31, 32]. Robotic gaze aversion (i.e., the intentional redirection away from the face of the partner in the interaction) is also perceived by humans as intentional and thoughtful, which can effectively shape the interaction [33]. Researchers have also investigated different gaze behaviors in avatars [34, 35] where inferred (from voice) gaze behavior enhanced the behavioral realism. It has also been shown that the duration of a gaze cue, in a social interaction setting, plays a significant role on the level of co-presence [36]. Previous studies have shown that, during verbal communication, active gaze behavior improves avatar liveliness and human-similarity [35–37]. For example, gaze dynamics (shifts, aversion, and fixation) can influence the sense of affiliation [38]. In another study, human gaze has been tracked to orient the avatar gaze in order to create eye-contact leading to the sense of awareness of others’ gazes in virtual interaction settings [39]. Moreover, responsive gaze behavior from an avatar can elicit in a human partner the feeling of being looked at [40]. Despite numerous studies on the realism of avatars [41, 42], and the realism of simulated gazes in virtual environments [35], little is known about the effects of avatar gazes in social motor coordination. In particular, it is unclear whether in joint action settings, avatars can effectively simulate natural gaze behavior, and whether human partners can benefit from it.

Similarity is believed to be an important factor for affiliation/attraction [43, 44]. Thus, it would be interesting to see if the same principle can be applied to the avatar-robot (or human-robot) interaction, where a different aspect of similarity—gaze cues in our case—can boost affiliation. To increase realism in animated avatars, several models of gaze have been proposed; see [45] as an example where the avatar head moves between poses according to the desired gaze behavior. To create human-inspired interactions, the avatar gaze has been programmed to be reactive to the human gaze that is tracked with wearable devices [46] or cameras [47]. Moreover, as the avatar’s hand was used for the mirror game, models suggested for human eye-hand coordination can be helpful in increasing behavioral similarity between avatars and humans. However, such proposed models in the literature are highly task-dependent; see [48] for search, [49] for sequential target contact, [50] for drawing, and [51] for rhythmical pointing tasks. Therefore, to keep the analysis simple, robust, and interpretable, we limited our gaze-hand model to a simple delay of 500*ms*, which is in line with previous findings in [13] and [52]. In order to check if similarity-affiliation effect persists in the case of motor coordination, we accompanied our experiment with a short questionnaire where participants’ opinions on human-similarity and on cooperation of the avatar are queried. We hypothesize that preceding movements of gaze helps the human partner with the action-prediction process which consequently improves the coordination and perception of human-likeness. Cross-checking the questionnaire results with the actual recorded performances enabled us to elaborate on these effects.

**Fig 1:**
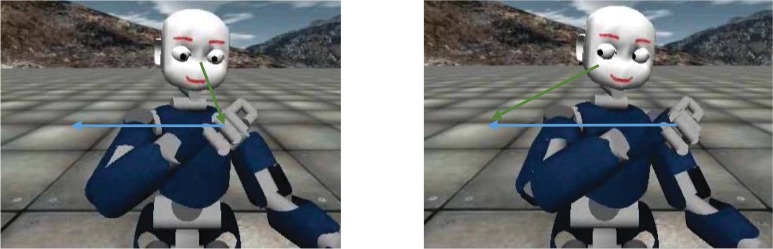
The simulated iCub robot. The robot is acting as the leader in the mirror game, generating random sinusoidal trajectories. (Left) the gaze is fixated on the hand. (Right) the gaze precede the hand. The blue arrows shows the next hand movement and the green arrows show the current gaze fixation point.

In this study, we investigate using an avatar, the role that gaze plays in socio-motor coordination. Producing structured and repetitive yet random motions, the avatar acts as the leader in the interaction and the participants are the followers. Based on the aforementioned evidence for the role of gaze direction in social interactions, we consider a human-avatar mirror game where the avatar provides the human follower with gaze cues indicating the direction-of-hand motion (i.e., the gaze precedes the hand motion). To have a control condition that can act as a baseline in our analysis, we use the case where the avatar does not provide the follower with a gaze cue; i.e., the gaze and hand moves synchronously, see Fig 1. A total of 344 trajectories (30*s* long each) were recorded and used for the analysis. To assess whether the participants exploited the gaze cues, the following metrics are used to quantify temporal coordination: (a) *reaction times*, using temporal errors at sharp changes in motion direction, and (b) *phase-frequency response*, using a decomposition of the dyad’s motion in frequencies. Frequency domain techniques provide more transparent analysis, as leader-follower coordination can be expressed by a set of phase relations in this domain. These techniques provide us with a better understanding of where and when in the motion the gaze cues improve the synchrony. We hypothesize that (1) participants would exploit gaze cues, marked by improvements in their coordinations and (2) the active gaze behavior for avatars/robots makes them seem more human-like to the human partners. In the next section, we present our methodology for investigating these hypotheses.

## Materials and Method

### Participants

We recruited 37 participants (26 male and 11 female) from the EPFL campus (Bachelor, Master’s, and PhD students). Their average age was 23.1 (4.7) [18–39] (values are presented in the form mean (standard deviation) [min-max]). Each participant took part in one session that lasted a maximum of 10 minutes. No inclusion/exclusion criteria were used for the recruitment and all participants successfully completed the session. As a consequence, no data had to be removed from the experiment. They also provided written informed consent to take part in this experiment.

### Apparatus

In this study, we used a computer-generated avatar that simulates the humanoid robot iCub [53], a 53-DOF humanoid robot as shown in Fig 1. In the experiment, the avatar is the leader and is programmed to produce a series of sinusoidal hand motions (different in terms of amplitude, frequency, and offset), following a virtual horizontal line orthogonal-to-sagittal plane. The parameters of the trajectories [offsets, amplitudes, frequencies, and random transitions] were hand-tuned based on human trajectories (studied in our previous work [52]), hence they display dynamics that are qualitatively close to human natural-dynamics. Randomness was added (to offset, amplitude, and frequency) to avoid that the human player learns the pattern of the motions and use this as a predictor. The head and eyes of the robot are controlled so as to generate the desired gaze behavior. The gaze direction is generated mostly by the eye movement, and the head movement was used to create a more natural and human-like behavior. In the *gaze cue* condition, the eyes precede the hand motion; the hand’s trajectory was used for the gaze, but with 500*ms* lag. In the *no-gaze cue* condition, the eyes are locked on the hand and move in synchrony with the hand, see Fig 1. In our analysis, this condition serves as the baseline for participants’ performances.

**Fig 2:**
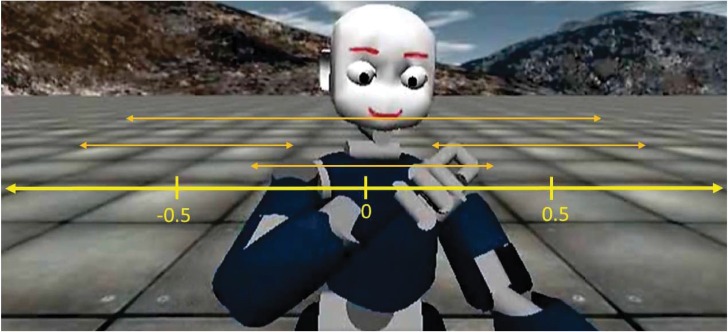
Patterns of movements. Modes of oscillations comprise random motions of the avatar’s hand. Three small oscillations (one to the left, center, right of the torso with amplitude of 0.3) and one large oscillation (amplitude of 0.7). Number of oscillations in each mode and transition to the next mode are random. The symmetric reachable range of the hand is scaled to [-1, +1], and it into the avatar’s coordinates.

To play the mirror game as the leader, we controlled the right arm of this robot. We used a standard inverse kinematics solver to control the motion of the 6 degrees of freedom of the right arm of the robot, so as to accurately follow the desired hand trajectory. In our inverse kinematics solver, we also considered human-like postures (motion of the shoulder and elbow). To use the robot as the leader in the mirror game, we controlled the position of the hand with a sinusoidal reference trajectory with stochastic parameters (random amplitude, offset, and frequency). We used random patterns in the motion to avoid that the human player learns the pattern of motions and uses this as a predictor; this keeps the gaze cue as a useful predictor during the interaction. In order to have this randomness in the avatar’s hand motions, we first scaled the hands reachable range to [−1, +1]. This reachable range, with respect to the body sagittal plane, is asymmetric. Then, we considered four modes of oscillation as depicted in Fig 2. Each mode has a different combination of offsets and amplitudes as follows:

$$\left[\begin{array}{c} offset\\ amplitude \end{array}\right]\in \left\{\begin{array}{c} \left[\begin{array}{c} 0\\ .3 \end{array}\right],\left[\begin{array}{c} -.5\\ .3 \end{array}\right],\left[\begin{array}{c} .5\\ .3 \end{array}\right],\left[\begin{array}{c} 0\\ .7 \end{array}\right] \end{array}\right\}$$ (1)

**Fig 3:**
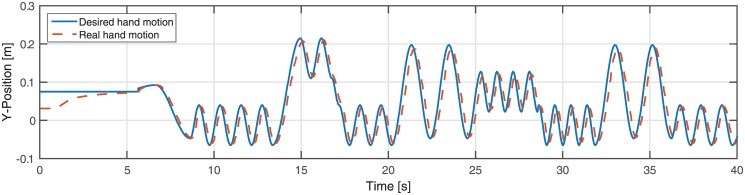
A sample of generated motion for the avatar’s hand. Tracking performance of the PD controller in this simulator is considered satisfactory. It is visible that the generated motion is composed of different modes (combination of offset and amplitude).

The number of oscillations in each mode is a random number between 2 and 5 (inclusive and uniform) except for the large oscillation where the number of oscillations is fewer (one or twice). Starting a mode, velocity of the oscillation is also selected randomly (1 or 1.3m/s) increasing the difficulty of the game. Moreover, upon completion, the next mode is randomly (and uniformly) chosen. This results in a random trajectory in each trial as shown in Fig 3.

The choice of parameters affects the level of difficulty of the game; switching quickly between different modes of oscillation results in fast and highly transitory motions which are harder to follow. By varying the parameters (speed and complexity of the motion) prior to the experiment, we adjusted the difficulty of the game to amplify the effects of gaze cues; at a higher level of difficulty, only relying on the hand motions does not result in a satisfactory tracking performance. Thus, we expected participants to pay attention to gaze cues and exploit this information throughout the game and, in particular, during the phases where the difficulty was the highest, specifically when the avatar changes direction of motion very rapidly. To avoid compounds due to unnatural dynamics of motion, we provided the avatar with motions that follows closely the typical dynamics of human hand motions in terms of range and frequency (studied in our previous work [52]). Fig 3 illustrates an example of such generated hand motions and the tracking performance of the controller.

To control the gaze, we used the default gaze inverse-kinematic solver provided by the iCub simulator [54]. In this solver, both head and eye movements are used to generate the gaze fixation point; 3 degrees of freedom for the eyes (azimuth, elevation, and vergence angles) and 3 degrees for the head (pitch roll and yaw angles). Parameters used to generate smooth and human-like gaze behavior are reported in S1 Table.

The head and eyes of the robot are controlled so as to generate the desired gaze behavior. The gaze direction is generated mostly by the eye movement, and the head movement was used to create a more natural and human-like behavior. In the *gaze cue* condition, the eyes precede the hand motion; the hand’s trajectory was used for the gaze but with 500*ms*. In the *no-gaze cue* condition, the eyes are locked on the hand and move in synchrony with the hand, see Fig 1. In our analysis, this condition serves as the baseline for participants performance.

As mentioned before, our experiment has two conditions. In the *no-gaze cue* condition, the eyes are locked on the hand and move in synchrony with the hand. This is illustrated in the first row of Fig 1, where the hand gaze receives the same desired trajectory. In the *gaze cue* condition, the gaze precedes the hand motion by 500*ms*, but only with respect to the offset of the oscillation as plotted in the second row of S1 Fig. It can be seen that the real gaze-trajectory differs from the desired one. This is due to the gaze controller being affected/perturbed by the hand motion. However, the leading behavior, which provides gaze cues, is preserved; the gaze moves sooner to the new offset and oscillates synchronously with the hand, and has a smaller amplitude.

**Fig 4:**
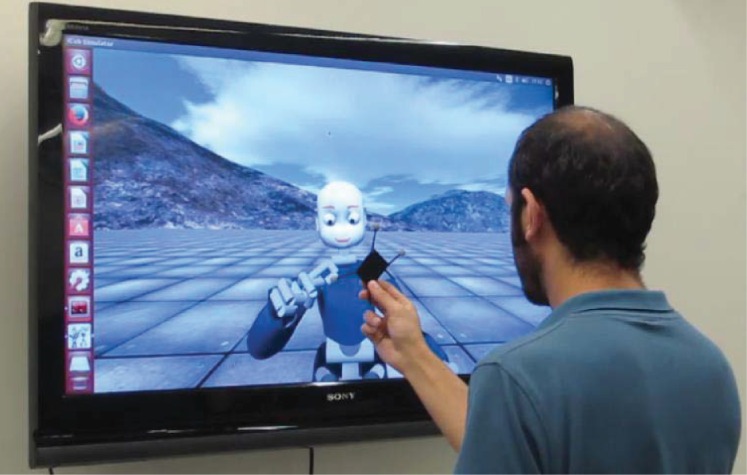
The experimental setup. The avatar is displayed on a big screen (46 inches). The avatar led the mirror game and the participant followed the avatar’s hand motions. The participant held a marker for motion tracking purposes.

In our experiment, participants were asked to follow the motion of the avatar, see Fig 4. To track the motion of the human’s hand, we asked the subject to hold a marker, which enabled us to track their motion using OptiTrack system [55] (120Hz for sampling rate, and accuracy of 0.1mm).

### Procedure

**Fig 5:**
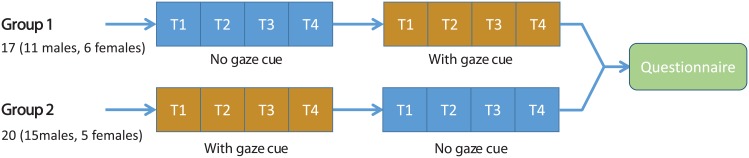
The protocol used for the experiment. Subjects were divided into two groups and participated in the experiment with a different ordering of conditions followed by a short questionnaire.

Each participant participated in both conditions. In order to remove the order effects, we divided the participants into two groups: one group was exposed to the “*no-gaze cue*” condition first, and the other was exposed to the “*gaze cues*” condition first. See Fig 5 for our experimental protocol. In each condition, subjects played four consecutive trials, each 30 seconds long. Having played in both conditions, the participants were asked to answer a short questionnaire. This led to a total of 344 recorded trajectories (30*s* long each) for the analysis. Upon completion of all the trials, we asked the participants five short questions about their impressions of the difficulty and realism (similarity to human behavior) of the avatar; see S2 Fig.

### Data Analysis

**Fig 6:**
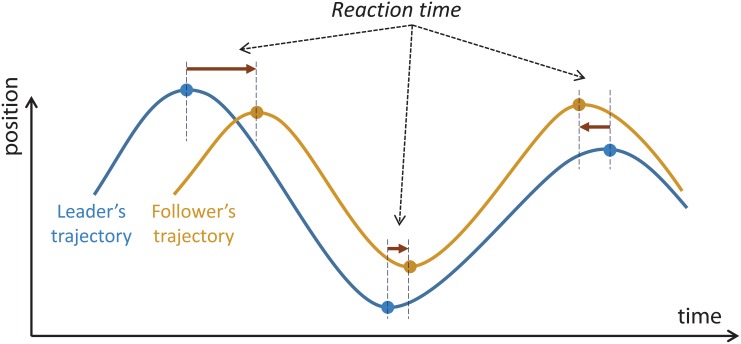
Reaction time analysis. Extraction of reaction time based on zero-velocity points in the leader and follower trajectories. In this conceptual example, we have positive reaction times (the leader/follower is leading/lagging) in the first two cases, and a negative reaction time (the leader/follower is lagging/leading) in the last case.

In our previous studies [52], we found that the human tracking performance can be captured by the temporal differences between the leader and the follower trajectories. Here we use the same measure; see Fig 6. For each set of leader-follower trajectories obtained from a trial, we calculate the temporal differences between the leader and the follower only across the peaks (i.e., zero-velocity points). The sign of the temporal difference shows whether the follower is leading or lagging. For each subject in a condition, we obtain a distribution for such temporal differences. We chose the average to compare the tracking performance across the two conditions, i.e., average reaction-time (RT). We refer to the within-subject RT contrast across the condition as RT improvement defined as

$$\Delta RT=RT_{n}-RT_{g}$$ (2)

where *RT*_*n*_ and *RT*_*g*_ represent the participants’ reaction times in “*no-gaze cue*” and “*gaze cues*” conditions respectively. A positive value for this variable shows that the participant had a better performance in the presence of the gaze cues.

To check the effect of gaze in more detail, we applied frequency-domain techniques. This allows for a more refined analysis where the leader-follower interaction is presented as a frequency-phase relation. This helps us to understand how gaze cues improve the coordination. A cross-wavelet transform was applied to the leader-follower trajectories by using a Matlab toolbox provided by [56]. In this transform, the Morelet wavelet with conventional temporal resolution (*σ* = 6) was used.

To pinpoint significant within-subject contrasts across the conditions, repeated measures ANOVA was performed. The reaction time, the perception of difficulty, and the perception of similarity are the three dependent variables which we measured in the two conditions; i.e., “*no-gaze cue*” and “*gaze cues*”. The condition and the order of the conditions are used as within-subject factors; i.e. independent variables. Moreover, a separate analysis included further the effect of age and gender were age was split into tree balanced groups as described in S2 Table.

## Results

We first present the results of our questionnaire. Then, we investigate the results obtained from the motion capture systems. Afterward, we crosscheck the subjects’ performances with their impressions reported in the questionnaire. Finally, we present the results acquired from the frequency-domain analysis of the recorded participants’ motions.

### Questionnaire Results

#### Cooperative and Natural Interaction by Using Gaze

**Fig 7:**
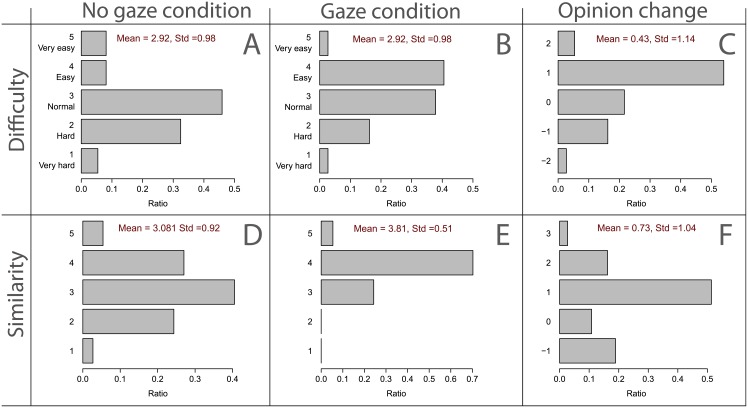
Distributions obtained from the answers to the questionnaire. (A) Difficulty in the “no gaze” condition. (B) Difficulty in the “gaze” condition. (C) Changes in the subjects’ opinion from the “no gaze” to the “gaze” condition. (D) Similarity to human behavior in the “no gaze” condition. (E) Similarity to human behavior in the “gaze” condition. (F) Changes in the subjects’ opinion form the “no gaze” to the “gaze” condition. In these plots, ratio is calculated by the number of participants in each level divided by the total number of participants.

Fig 7 summarizes the response distribution for the first four questions of the questionnaire. Fig 7A shows that in the absence of gaze, most of the subjects found it slightly difficult to follow the avatar. whereas, Fig 7B shows that, in the presence of gaze, following the avatar is perceived as rather easy. Fig 7C shows how presence of gaze cues affected participants’ opinion on the level of difficulty. The majority of subjects (60%) perceived the mirror game as easy (by either 1 or 2 steps) in the *gaze cues* condition; see Fig 7C. The analysis of variance shows that opinions are significantly shifted toward low difficulty [*F*(1, 35) = 5.478, *p* = 0.025]. No significant effects were detected due to age, gender, and the order of the conditions; see S3 Table for more details. The second row of Fig 7 shows subjects’ responses to the question about how similar they found the robot’s behavior compared to human behavior. Fig 7D shows a bell-shaped distribution for similarity index in the absence of gaze whereas Fig 7E shows a skewed distribution in the presence of gaze implying a high similarity to human behavior when the avatar uses its gaze actively. Fig 7F illustrates how presence of gaze cues affected participants’ opinions on the level of realism. A majority of subjects (71%) perceived the avatar as more human-like (by either 1, 2, or 3 steps) in the *gaze cues* condition; see Fig 7C. The analysis of variance shows that opinions significantly shift toward high realism [*F*(1, 35) = 17.897, *p* = 0.000]. No significant effects were detected due to age, gender, and the order of the conditions; see S3 Table for more details. In summary, Fig 7 shows that use of gaze cues made the interaction easier, and elicited the avatar to be perceived as more human-like and realistic.

#### Correlation Analysis Between Cooperation and Realism

To determine if perception of difficulty (cooperative behavior) and human-likeness (realism) are correlated, we computed a contingency table, see S4 Table. This table is computed based the participants’ opinions about their performances in the *gaze cues* condition compared to the *no-gaze cue* condition. S4 Table shows that a majority of participants (sum of diagonal elements: 53%), who found the avatar more realistic in the presence of gaze cues, also found the interaction easier. However, no significant dependency between difficulty and realism was detected using Spearman’s correlation test in this table.

### Motion Capture Results

#### Reaction Time

**Fig 8:**
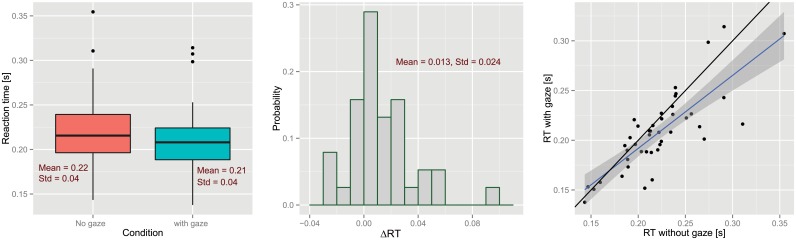
Overall analysis of the recorded motions. (Left) Boxplots of subjects’ reaction times in each condition. (Center) histogram of Δ*RT*. (Right) RT in the *gaze cues* condition vs. RT in the *no-gaze cue* condition. Each dot represents a participant. Black line is the unity line and the blue line in the result of the linear regression.

Now, we turn to the objective and quantifiable results on the effect of gaze on the subjects’ tracking performances. To this end, we analyzed the data on the relative velocity of participants and the avatar’s hand motions. As mentioned before, the tracking performance of each participant is measured by the average of absolute temporal error (so-called reaction time, or in short RT). Therefore, for each participant, we compute the RT for both *no-gaze cue* and *gaze cues* conditions. To contrast the two conditions, we take the difference between the RT in each case (Eq 2), which we name “Improvement in RT”. Fig 8 shows the overall results of this analysis.

Fig 8(Left) shows the boxplots for reaction times in each condition where participants, on average, showed faster reactions with gaze cues than without. The analysis of variance shows a significant improvement in reaction times due to the gaze cues [*F*(1, 35) = 9.445, *p* = 0.004]; see S3 Table for more details. Moreover, a marginally significant effects due to age was detected [*F*(2, 32) = 2.996, *p* = 0.064]. The post-hoc analysis showed that the old participants, compared to the young ones, have a significantly higher RT improvement; see S5 Table and S3 Fig for more details. Fig 8(Center) shows the distribution of Δ*RT*. The results of the Wilcoxon test suggests that the average of this distribution (13ms) is significantly greater than zero. The last subplot, Fig 8(Right), shows the performance of each individual change in the presence of the gaze cue. The black line indicates the unity line (the null hypothesis). As can be seen, the data is skewed to the favorable side of this line (alternative hypothesis). The blue line illustrates the linear regression of the data. The slope of this regression implies that individuals with lower performances (higher RT in the “no gaze” condition) can benefit more from gaze cues.

#### Frequency-Phase Profile

**Fig 9:**
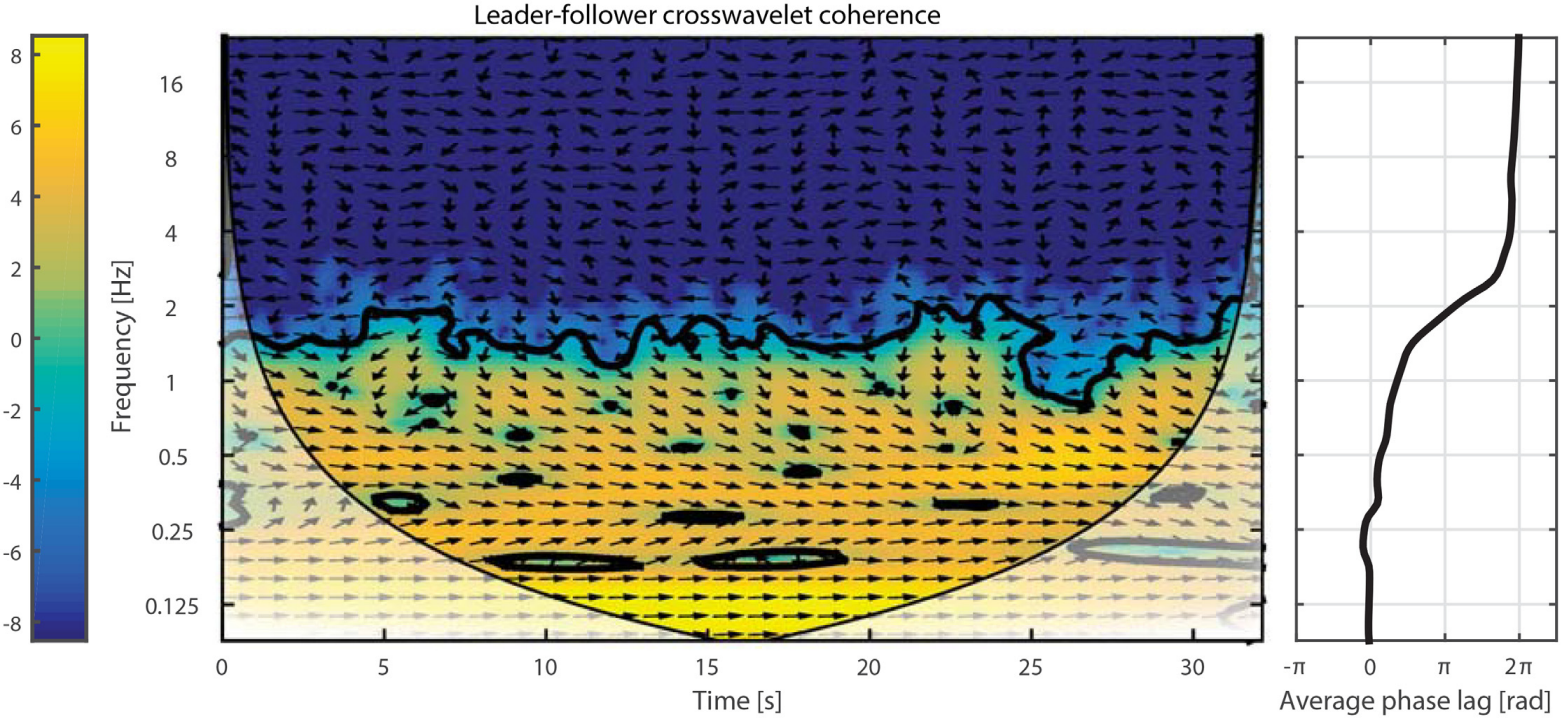
Cross-wavelet analysis. Right: Cross-wavelet coherence between the leader and the follower in one of the trials. Power of frequency components at each time is color coded; i.e., blue/yellow for weak/strong components, respectively. Moreover, the arrows indicate the leader-follower phase relation for each frequency over time. Left: Average phase-lag for each frequency extracted from the main plot.

Thus far, for our analysis, we used a metric based on the computation of zero-velocity points only. Although this metric provides a good estimation of the reaction time and enables us to put forward significant differences across the conditions. However, it does not provide an assessment for the different aspects of joint action; i.e., action prediction, temporal coordination, and joint planning. A decomposition of the avatar and human motions in the frequency domain, using wavelet analysis, offers powerful tools for attaining such quantitative assessments. By using wavelet analysis [57], the leader-follower interaction can be transformed into time-frequency space where the temporal correspondence is easier to detect compared to the reaction time analysis. For this purpose, we use the Matlab Wavelet Coherence toolbox provided by [56]. The results of cross-wavelet coherence for one of the trials are illustrated in Fig 9.

In cross-wavelet coherence, each point at a certain time and frequency has two components: power and angle. The power, which is color-coded in the figure, shows the strength of that frequency at that moment. The angle, however, shows the lag between the leader and the follower. The arrows, pointing to the right, indicate a perfect synchrony, whereas arrows tilting upward/downward show a leading/lagging behavior in the follower; upward/downward arrows signify 90 degree phase lead/lag. To quantify the temporal correspondence, we extracted the average phase-lag at each frequency; see Fig 9(Right). We observe that, in low frequencies, there is a satisfactory synchronization that deteriorates as frequency increases. There is an interesting point when the graph passes 90 degree, i.e., an asynchronous interaction. Similar to linear filters, this frequency (2Hz in this example) can be considered as the *bandwidth* of interaction; i.e., a frequency beyond which the synchronous interaction cannot be maintained. Moreover, after a certain frequency, the estimation of phase lag is not reliable as the power of that frequency drops in the cross-wavelet coherence plot.

**Fig 10:**
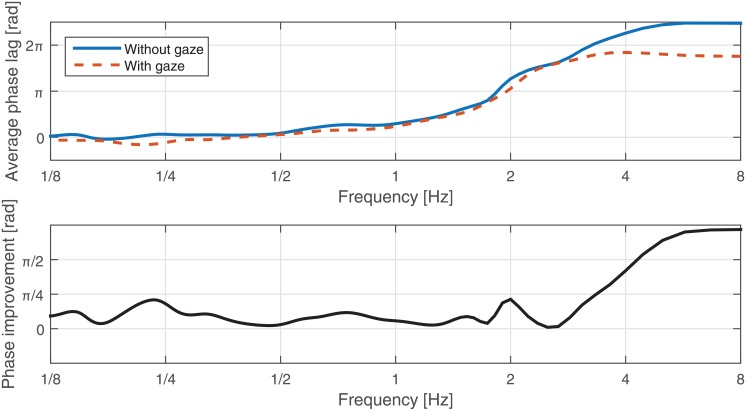
Frequency-phase profile. Top: Average phase-lag vs. frequency of one of the participants in both conditions; with and without gaze. Bottom: Phase improvement vs. frequency of one of the participants due to the presence of the gaze cues.

The average phase-lag can be extracted for each subject for the two conditions, i.e., with and without gaze. Such graphs, for one of the subjects, are plotted Fig 10. It can be seen that, for both cases, synchrony reduces as frequency increases. However, the interaction has a lower lag for each frequency in the presence of the gaze. This can be assessed easier by looking at the difference of two graphs in the lower plot in Fig 10. This plot clearly shows that, for this participant, the presence of the gaze improved the interaction over all frequencies.

**Fig 11:**
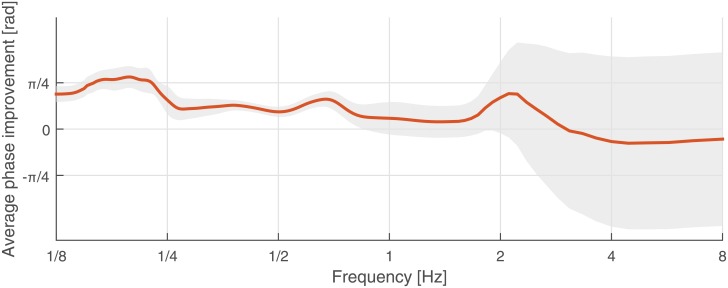
Effect of gaze on the synchrony of the interaction across frequency (averaged over all subjects). The red graph indicates the average improvement due to the gaze cues. Gray area indicates the scaled 95% confidence intervals.

We applied this procedure to all participants and studied the average behavior that is plotted in Fig 11. Investigating the 95% confidence interval does not show a significant improvement (with zero improvement as the null hypothesis). However, scaled standard deviations are plotted for comparison across the frequency spectrum. As mentioned before, the average phase for high frequencies is not reliable, which, in this figure, results in wide intervals. It can be seen that improvements take place in three different regions. Interestingly, each region accounts for a different underlying process in joint actions. These processes are as follows:

**Action prediction**: low-frequency region (1/8 − 1/4*Hz*) accounts for the variation of the offset in the motion; see Fig 3. By providing a gaze cue to the next location of the oscillations, the avatar improves the synchrony in the interaction in this region. Therefore, gaze affects the joint action by improving the action predication process.
**Action coordination**: mid-frequency region (1/2 − 1*Hz*) accounts for the oscillatory motions. The improvement in this range supports the hypothesis that, in the *gaze cues* condition, the follower can synchronously follow one mode of oscillation, which has a random number of repetitions, until the next gaze cue. Therefore, gaze affects the joint action by improving action coordination.
**Task sharing/Joint planning**: high-frequency region (around 2Hz) accounts for fast and transitory motions. The improvement in this region shows that faster synchronous interactions can be sustained in the presence of the gaze. The human-follower has more confidence in initiating these fast motions, as if the task/leadership is shared between the human subject and the avatar. Therefore, gaze affects the joint action by introducing joint planning and task sharing. However, compared to the previous regions, this result is not reliable due to the wider confidence intervals.

### Consistency Between Participants’ Perceived and Actual Performance

**Fig 12:**
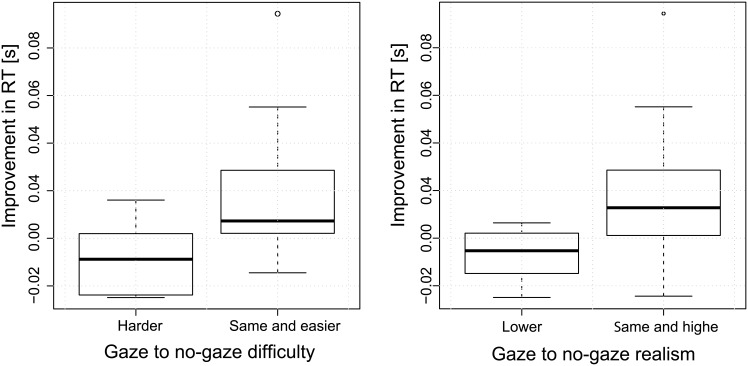
Participants’ actual performance vs. their perception. Boxplots of Δ*RT* for the participants who found it (Left) harder to follow with gaze cues compared to the rest of the participants, and (Right) less human-like with gaze cues compared to the rest of the participants.

To determine whether the participants’ actual performances are consistent with their impressions, we analyzed their reaction times with respect to their responses in the questionnaire. Fig 12(Left) compares RT improvements (due to the gaze) for the two groups: (1) the participants who found it harder to follow the avatar with gaze cue, (2) the rest of participants. The ANOVA reveals that these two groups are significantly different [*F*(1, 34) = 5.495, *p* = 0.025]; see *Model I* of S6 Table for more details. This means that participants who stated that it is harder to follow the avatar in the presence of the gaze cues, actually had a slower reaction time in the *gaze cues* condition.

Crosschecking the Δ*RT* with the results for realism from the questions reveals interesting facts: The participants who found the presence of gaze cues less human-like have significantly [*F*(1, 34) = 6.084, *p* = 0.019] lower performances in the *gaze cue* condition; see Fig 12(Right) and *Model II* of S6 Table in the Appendix for more details. Based on this analysis, we can infer that the sense of realism and cooperation (level of difficulty) are related; i.e., cooperation contributes to affiliation and vice versa.

In this work, we did not monitor explicitly the gaze of the participants. Incorporating eye trackers [58] and monitoring the subjects’ shifts of visual attention could contribute to a finer analysis of the pattern of attention. In our study, such monitoring could provide information on when the human partners pays attention to the robot’s face versus to the robot’s hand. However, we used a questionnaire to assess how participants managed to divide their attention between tracking the robot’s hand and looking at the robot’s gaze; a five steps rating system (i.e., very easy, easy, normal, hard, very hard). On average, participants found it easy to divide their attention between the hand and the gaze of the avatar; see S4 Fig for more details. No significant effect was detected for this factor on the RT contrast in the two conditions; see *Model III* of S6 Table in the Appendix for more details. However, participants who found it very easy, or easy to divide their attention had a faster RT in the *gaze cue* condition [*F*(1, 34) = 3.425, *p* = 0.073]; see S7 Table and S5 Fig for more details.

## Discussion

The embodiment of artificial agents plays an important role in their interactions with human partners. Many works in the literature on social robotics explore this feature. For example, the presence of robotic platforms has been considered a key element in evaluating therapy in the case of autism spectrum disorders [59]. Moreover, another recent study [60] has shown that a robotic referential gaze leads human partners to take the robot’s visual perspective. We share the same belief that embodiment can enhance the sense of affiliation. However, it is interesting to see that in this study, a gaze of a simulated robot on a screen can still elicit a sense of realism in the human partner. Replicating the same experiment using the humanoid robot, the iCub, in comparison with the avatar case, is an interesting investigation where we can study the difference between simulated and real platforms in the context of social robotics.

In this study, we used a simple model for eye-hand coordination, which does not reproduce the exact dynamics of eye-arm coordination found in humans. We learned that even such simple behavior helps the human partner with the action prediction process, and consequently improves the coordination and the perception of human-likeness. However, modeling more realistic eye-hand coordination for avatars might boost the behavioral realism and increase affiliation [45–48]. For avatars, reactive gaze behavior to the human gaze can also potentially enrich their realism [40]. However, reaching a robust statistical conclusion in face of such a complex behavior of the avatar requires more thorough experimental design with a larger sample size. In this preliminary work, we benefited from our simple gaze model. We reached the robust and interpretable results that enabled us to elaborate on effects of gaze on joint action and realism of computer simulated avatars.

These findings may support the design of similar games for studying deficiencies in the ability to interpret other people’s gaze, as displayed by individuals suffering from schizophrenia and autism spectrum disorders (ASD) [61–64]. Interpersonal synchrony provides an important foundation for social interaction, in which recent studies suggested that people suffering from schizophrenia and ASD also have deficits in motor coordination [65–68]. A recent study in schizophrenia found a causal relationship between impaired attention toward gaze orientation and deficits in theory of mind [63]. The version of the mirror game offered in our study, in which gaze is used as an active cueing device, could serve to design therapeutic games whereby patients are encouraged to process gaze information in order to increase motor synchrony during interactions. Improving interactional synchrony in schizophrenic patients, when engaged in dyadic games with a healthy partner, is shown to be beneficial for the patient and partner alike, as it also increases the motivation and sense of affiliation in the healthy partner [66]. Previous studies have already shown that schizophrenia patients can benefit from attentional-shaping procedures displayed by a therapist, to enhance neurocognition and functioning [69–71], or being instructed to pay more attention to facial areas that contain information about a displayed emotion to enhance emotion recognition [72]. However, the use of an avatar for therapy in place of a human is advantageous in that the avatar provides a consistent and reliable feedback/behavior without the presence of a therapist.

## Conclusion

In this study, we have tested whether, in a human-avatar joint action, an avatar gaze behavior can improve coordination. We used the mirror game paradigm where the human subject imitates the hand motions of a animated avatar. To test our hypotheses, we implemented a simple gaze behavior where an avatar provides a human subject with task-relevant cues. In a within-subject study, we recorded the performance of participants in the presence and absence of gaze cues. We assessed the avatar’s realism and cooperation by a post-hoc questionnaire. Our main result shows that gaze cues significantly improve participants’ reaction times to the avatar’s movements. A wavelet analysis of the interactions provided us with a better understanding of different underling aspects/processes reported for joint actions. Frequency-domain techniques helped us to model the follower’s behavior as a frequency-dependent-phase relation that, compared to time domain analyses, is easier to interpret. We learned that, in a joint action, the leader’s gaze cues helps the follower with action prediction, action coordination, and task sharing. The results of the questionnaire showed that participants perceived the avatar’s gaze cues behavior not only as cooperative, but also human-like and realistic. Moreover, we observed that participants perception of similarity and cooperation is correlated with their performance in the game. This suggests that human-similarity, cooperativeness, and the sense of affiliation toward avatars, are highly interlinked. The results of this study will help us design computer-assisted cognitive-remediation therapy for pathologies with abnormal gaze and motor behavior such as schizophrenia.

## Limitations

To best of our knowledge, this study is the first to investigate the effect of avatars’ gaze behavior on social motor coordination. Thus, the results must be considered as exploratory where we used a straightforward gaze model in a simple interactional framework (i.e., the mirror game). For further enhancement of avatar realism, future work should explore more sophisticated gaze models; e.g., models inspired by human behavior. It is also interesting to perform the experiment using the humanoid robots to investigate if gaze effects can be generalized to other non-human agents. In this study, we used two metrics: reaction time and frequency-dependent-phase. Both metrics captured the beneficial effects of gaze cues. We believe that the second metric was introduced for the first time in this study. Due to a higher effect size in this metric (the entire frequency domain), however, a larger sample size is required to reach substantial statical power in order to draw significant conclusions. Future studies should consider eye tracking to correct for the participants’ level of attention to the avatar’s gaze in the statistical inferences.

## Supporting Information

S1 Fig An example of desired trajectories for the avatar’s hand and gaze in two conditions. (EPS)

S1 Table Parameters used in the iCub gaze controller. (EPS)

S2 Fig The questionnaire used in this study. (EPS)

S2 Table The split performed on age for the ANOVA analysis. (EPS)

S3 Fig The reaction time improvement due to the gaze cues across age. The ANOVA analysis in S5 Table showed that the first group (*Low*) and the last group (*High*) are significantly different.
(EPS)

S3 Table The results of the Repeated Measures ANOVA. In each condition (i.e., *gaze cue* and *no-gaze cue*), the three different measurements done are: 1) the reaction time, 2) the perception of the difficulty of the game, and 3) the perception of the human-similarity. In *Model I*, the effects of conditions and the order of the conditions are studied. In *Model II*, the effects of age and gender are also investigated.
(EPS)

S4 Fig Participants’ attentional workload. The distribution obtained from the answers to the questionnaire concerning the division of attention between avatar’s gaze and hand.
(EPS)

S4 Table Correlation between cooperation and realism. Contingency table for effect of gaze cues on participants’ opinion on the difficulty of the interaction and the realism of the avatar.
(EPS)

S5 Fig The RT in *gaze cue* condition vs. attention. The *RT*_*g*_ distribution of participants who found it hard to divide their attention between the avatar’s gaze and hand compared to the rest of the participants. The ANOVA analysis in S7 Table showed that the difference in these distributions is significant.
(EPS)

S5 Table The post-hoc test for age. The post-hoc test for the detected effect of age on the reaction time in S3 Table. The multiple comparisons are done based on LSD method. The corresponding distributions are plotted S3 Fig.
(EPS)

S6 Table Crosschecking the result of the motion capture (i.e., RT) with the result of the questionnaire using repeated measures ANOVA. In *Model I*, the effect of perception of difficulty on RT is studied. *Diff_dummy* is 0 for the participants who found it harder to follow the avatar with gaze cue, and 1 for the rest of the participants. In *Model II*, the effect of perception of similarity on RT is studied. *Sim_dummy* is 0 for the participants who found the presence of gaze cues less human-like, and 1 for the rest of the participants. In *Model III*, the effect of attention load on RT is studied. *Sim_dummy* is 1 for the participants who found it very easy, or easy to divide their attention between the avatar’s gaze and avatar’s hand, and 0 for the rest of the participants.
(EPS)

S7 Table The effect of attention of the RT. The results of the univariate ANOVA to study the effect of attention on the RT in the *gaze cue* condition. *Att_dummy* is 1 for the participants who found it very easy, or easy to divide their attention between the avatar’s gaze and avatar’s hand, and 0 for the rest of the participants; see S5 Fig. Moreover, Levene’s test indicated equal variances [*F*(14, 22) = .743, *p* = 0.713].
(EPS)

S8 Table Equality of variances. The Levene’s test of equality of error variances for *Model I* and *Model II* presented in S3 Table. For both models df1 = 28 and df2 = 8.
(EPS)
